# Exercise Metabolomics Reveals Intensity-Dependent Metabolic Responses Associated with Cardiorespiratory Fitness in Adults with Type 1 Diabetes

**DOI:** 10.3390/metabo16090687

**Published:** 2026-09-18

**Authors:** Olivia M. McCarthy, Clara Solà, Chloe Nicholas, Sandra Tawfik, Merete Bechmann Christensen, Signe Schmidt, Kirsten Nørgaard, Richard M. Bracken, Marietta Kokla

**Affiliations:** 1 Copenhagen University Hospital, Steno Diabetes Center Copenhagen, Borgmester Ib Juuls Vej 83, 2730 Herlev, Denmark; 2 Applied Sport, Technology, Exercise and Medicine Research Centre, Faculty of Science and Engineering, Swansea University, Bay Campus, Fabian Way, Swansea SA1 8EN, UK; 3 Department of Clinical Medicine, Faculty of Health and Medical Sciences, University of Copenhagen, Blegdamsvej 3B, 2200 Copenhagen N, Denmark; 4 Health Technology and Solutions Interdisciplinary Research Institute, Faculty of Science and Engineering, Swansea University, Bay Campus, Fabian Way, Swansea SA1 8EN, UK

**Keywords:** cardiorespiratory fitness, energy metabolism, exercise physiology, metabolomics, type 1 diabetes

## Abstract

**Highlights:**

**What are the main findings?**

• Exercise intensity is a key determinant of the circulating metabolome, with dynamic changes in metabolites related to glycolysis, the tricarboxylic acid cycle, and purine metabolism occurring predominantly at higher physiological workloads.
• Higher cardiorespiratory fitness was associated with greater exercise-induced changes in circulating glycolysis-related and tricarboxylic acid-cycle metabolites.

**What are the implications of the main findings?**

• These findings provide insight into the intensity-dependent metabolic responses to exercise in adults with type 1 diabetes.
• They support the application of metabolomics as a complementary tool for understanding exercise capacity in adults with type 1 diabetes.

**Abstract:**

**Purpose**: To characterize plasma metabolomic responses to maximal exercise and their associations with cardiorespiratory fitness (CRF) in adults with type 1 diabetes. **Methods**: In this exploratory pooled secondary analysis, 21 adults (67% female; age, 51.6 ± 13.2 years; HbA1c, 67.5 ± 7.0 mmol/mol) completed a cycle-ergometer test to exhaustion; metabolomic data were available for 20. Plasma collected at rest, anaerobic threshold (AT), peak exercise, and recovery underwent targeted and untargeted gas chromatography–mass spectrometry profiling. Peak oxygen uptake (V̇O_2_peak) quantified CRF. Linear mixed-effects models adjusted for age, sex, HbA1c, and diabetes duration. **Results**: At the AT, L-(+)-lactic acid and 2-hydroxyisobutyric acid increased relative to rest (both *q* ≤ 0.010). Peak exercise produced changes in glycolytic, tricarboxylic acid (TCA)-cycle, and purine metabolites, with enrichment of the TCA cycle and nucleotide metabolism. TCA-cycle enrichment persisted during recovery. In exploratory interaction analyses, higher V̇O_2peak_ was associated with increases in L-(+)-lactic acid at AT (β = 0.032, *q* = 0.040) and peak exercise (β = 0.031, *q* = 0.024), and in malic acid at peak exercise (β = 0.063, *q* = 0.010) and recovery (β = 0.061, *q* = 0.015). **Conclusions**: These preliminary findings suggest the presence of intensity-dependent and fitness-associated metabolic responses to exercise in adults with type 1 diabetes. Given the small sample size, particularly for interaction analyses, these findings require confirmation in larger independent cohorts.

## 1. Introduction

Cardiorespiratory fitness (CRF) is recognized as one of the strongest predictors of all-cause and cardiovascular mortality [1,2,3,4]. Compared with healthy individuals, adults with type 1 diabetes exhibit significantly lower CRF [5,6,7]. Although multifactorial, this impairment has primarily been associated with chronic hyperglycemia and the presence of diabetes related complications [7,8,9,10]. While informative, these long-term clinical markers provide limited insight into the metabolic processes underlying CRF.

Physiologically, CRF reflects the integrated capacity of the pulmonary, cardiovascular and skeletal muscle systems to transport and utilize oxygen for adenosine triphosphate (ATP) production [4]. During maximal exercise, skeletal muscle can increase ATP turnover by approximately 1000-fold despite storing minimal ATP. Meeting these energetic demands requires tightly coordinated contributions from glycolysis, the tricarboxylic acid (TCA) cycle and oxidative phosphorylation to sustain muscular work [11,12]. Characterizing these responses therefore offers an opportunity to better understand the biological processes underpinning exercise capacity in type 1 diabetes.

Recent developments in mass spectrometry-based metabolomics enable comprehensive in vivo profiling of these dynamic bioenergetic responses, providing physiological insight beyond that offered by conventional clinical markers [13,14,15]. Though limited in number, previous studies applying metabolomics around acute physical exercise in those with type 1 diabetes have demonstrated distinct metabolomic responses compared with healthy controls [16], as well as differences according to exercise modality [17], glycemic conditions [18] and residual beta-cell function [19]. However, these studies have largely relied on fixed exercise protocols or resting metabolomic profiling [16,17,18,19], precluding an understanding of how the metabolome evolves across increasing exercise intensity.

Given the rapid turnover of metabolic intermediates during exercise, serial sampling is essential to capture the temporal regulation of energy metabolism. Furthermore, although resting metabolomic signatures have demonstrated considerable prognostic value for future disease risk [20] and can distinguish individuals with type 1 diabetes according to CRF status [19], how these signatures evolve dynamically during exercise remains unclear.

Therefore, the aim of this study was to characterize the plasma metabolome throughout maximal exercise testing and determine the metabolic signatures associated with CRF in adults with type 1 diabetes.

## 2. Materials and Methods

### 2.1. Study Design and Ethical Governance

The present study was an exploratory, pooled secondary analysis of screening data from two randomized crossover clinical trials (Clinical Trials Register; ClinicalTrials.gov identifiers: NCT05133765, NCT05134025) with identical cardiopulmonary exercise test (CPET) protocols performed at the screening visits [21,22]. The trials were conducted according to the Declaration of Helsinki, and all procedures were approved by the National Research Ethics Committee of the Capital Region of Denmark (H-19029796). All volunteers received written and verbal information about the study and provided written informed consent for their participation.

### 2.2. Participants

Eligible participants were aged 18–75 years, had type 1 diabetes for ≥2 years, had HbA1c ≥ 58 mmol/mol (7.0%), had used an insulin pump for ≥12 months, had used a continuous or intermittently scanned glucose-monitoring system for ≥6 months and had used insulin aspart for ≥1 week.

Main exclusion criteria: females who were pregnant or breastfeeding; use of glucose-lowering medications (other than insulin), corticosteroids and/or other drugs affecting glucose metabolism during the study period or within 30 days prior to study start; daily use of acetaminophen; alcohol or drug abuse; and conditions for which an HbA_1c_ target below 53 mmol/mol (7.0%) was considered.

Twenty-one participants completed the CPET and were included in the clinical description. Metabolomics and plasma glucose analysis included 20 participants. The available analytic samples were determined by the number of eligible participants from the two parent trials who completed the common screening protocol and had available metabolomic data.

One metabolomic dataset was unavailable because of a technical failure during mass-spectrometric analysis.

### 2.3. Cardiopulmonary Exercise Testing

Participants abstained from caffeine for 12 h and from alcohol and exercise for 24 h before testing, maintained their usual insulin regimen and consumed a meal ≥ 2 h before the visit. Before exercise, contraindications were assessed according to American College of Sports Medicine guidelines [23] and an intravenous cannula was inserted for venous blood collection.

Participants performed a graded CPET on a workload-controlled cycle ergometer. The protocol comprised 3 min seated rest, 3 min warm-up at 20 W, 1 min incremental workload stages until volitional exhaustion, 3 min active recovery at 20 W and 3 min passive recovery [24]. The mean ± SD incremental exercise duration was 9.93 ± 3.2 min.

Breath-by-breath gas exchange was measured using a Vyntus ONE analyzer (Vyaire Medical GmbH, Höchberg, Germany), and heart rate was recorded continuously by chest-belt telemetry. Data were exported at 5 s intervals and averaged over 30 s. The anaerobic threshold (AT) was determined using the Exercise Threshold App (www.exercisethresholds.com) [25]. V̇O_2peak_ was defined as the highest 30 s oxygen-uptake value before peak workload and was used because an oxygen-uptake plateau was not identifiable in all participants.

Venous blood was collected at rest, every 3 min during incremental exercise, at volitional exhaustion and 6 min after exhaustion. We selected the venous sample collected closest in time to the individually determined AT for analysis. Among participants with both timing measurements available (n = 19), the modeled AT occurred at 11.47 ± 2.42 min, and the corresponding blood sample was collected at 11.21 ± 2.80 min. There was no evidence of a systematic difference between the modeled AT and blood-sampling times (*p* = 0.181). At the individual level, the mean absolute timing difference was 43.2 ± 27.3 s (median, 45.0 s; IQR, 19.8–62.4 s). Fourteen of the 19 samples (73.7%) were collected within 60 s of the modeled AT, and all samples were collected within 90 s of the modeled AT.

Complete eligibility, pre-exercise standardization, exercise-testing and blood-sampling procedures are provided in the Supplementary Materials (Supplementary S1–S6, Supplementary Figure S1).

### 2.4. Metabolomic Analyses

Targeted and untargeted plasma metabolomic profiling was performed using gas chromatography–mass spectrometry. Methanol-extracted samples containing isotopically labeled internal standards were derivatized by methoximation and trimethylsilylation and analyzed using an Agilent 7250 GC/Q-TOF system coupled to an Agilent 8890 gas chromatograph. Targeted and untargeted data were processed separately using MassHunter Quantitative Analysis and MS-DIAL, respectively, and subsequently merged. When both workflows annotated the same metabolite, the targeted signal supported by an analytical reference standard was retained. Features were normalized to the relevant internal standards and subsequently quantile normalized across samples to harmonize their empirical abundance distributions. Features were retained when the relative standard deviation was <0.20 and the D-ratio was <0.40 [26]. Complete details of sample preparation, analytical conditions, metabolite identification, quality control, normalization and data integration are provided in Supplementary S7–S13.

### 2.5. Statistical Analysis

The combined dataset contained 164 metabolites measured in 80 biological samples from 20 participants across four exercise workloads, corresponding to 13,120 expected metabolic measurements. Metabolite values were log_2_-transformed [log_2_(x + 1)] [27]. Thirty-four metabolite measurements (0.259%) were missing overall, all for cysteine (34/80; 42.5%); the other 163 metabolites were complete. The missing cysteine values remained missing in the final modeling matrix, and the cysteine-specific mixed-effects models used available observations. Clinical and plasma glucose values were not imputed. Descriptive statistics are presented in Table 1 and Supplementary Table S1A,B.

No separate a priori sample size or power calculations were performed for this exploratory secondary metabolomic analysis because the analytic samples were fixed by the number of eligible participants with available data from the two parent trials. Although each participant contributed measurements at four exercise stages, the dataset comprised 20 independent participants.

A separate linear mixed-effects model (LMM) was fitted for each of the 164 metabolites; the metabolites were not included simultaneously as predictors in a single model. Each model included metabolite abundance as the outcome and exercise workload as a categorical fixed effect across four workloads (rest, AT, peak exercise, and recovery). Rest was specified as the reference, and participant ID was included as a random intercept to account for the correlation among repeated measurements from the same participant. Model 0 examined the unadjusted effect of exercise workload. Model 1 was adjusted for age, sex, HbA1c, and diabetes duration. Model 2 and Model 3 examined workload interactions with HbA1c and resting glucose, respectively. Model 4 examined the independent association of V̇O_2peak_ with metabolite abundance, and Model 5 examined workload × V̇O_2peak_ interactions. Continuous interaction variables were mean-centered (Supplementary Table S2A).

All metabolomic analyses were considered exploratory. In particular, Models 2, 3, 5 were regarded as hypothesis-generating interaction analyses because HbA1c, resting glucose and V̇O_2_peak were participant-level potential effect modifiers measured in only 20 independent participants.

Models were fitted by maximum likelihood. Denominator degrees of freedom and two-sided fixed-effect *p*-values were calculated using Satterthwaite’s approximation, and 95% Wald confidence intervals were reported. *p*-values were adjusted using the Benjamini–Hochberg false discovery rate (BH-FDR) procedure separately within each model term, with *q* < 0.05 considered statistically significant. The BH-FDR procedure addresses multiple testing across metabolites but does not address potential imprecision or instability arising from the small participant-level sample size.

For metabolites with significant workload × V̇O_2peak_ interactions in Model 5, predicted workload-minus-rest responses were calculated at the 10th and 90th percentiles of V̇O_2peak_. Other continuous covariates were held at their means, and sex was held at the reference category (female; Supplementary Table S2B).

Changes in plasma glucose concentrations across workloads were evaluated using one-way repeated-measures ANOVA, followed by paired *t*-tests with Holm adjustment (Supplementary Tables S3 and S4). Exploratory Pearson correlations assessed associations between clinical and exercise characteristics and workload-specific metabolite changes relative to rest (ΔAT–Rest, ΔPeak–Rest and ΔRecovery–Rest). Each correlation analysis was restricted to metabolites with a significant Model 1 effect for the corresponding workload contrast. Correlation *p* values were not adjusted for multiple testing (Supplementary Table S5).

All 164 metabolites included in Model 1 were assigned to one of nine mutually exclusive broad metabolic categories using Kyoto Encyclopedia of Genes and Genomes (KEGG) annotations where available, supplemented by predefined biochemical classification. Metabolites without a specific category assignment were classified as other/unclassified. Metabolic-category over-representation was assessed separately for each workload contrast using one-sided hypergeometric tests, with all 164 metabolites constituting the background universe. *p* values were adjusted across the nine categories separately within each workload comparison using the BH-FDR procedure (Supplementary Table S6).

Additional details of data preprocessing, model implementation, prediction analysis, correlation analyses, and metabolic-category over-representation are provided in Supplementary S14–S25.

### 2.6. Software

Statistical analyses were performed in R version 4.3.2 (R Foundation for Statistical Computing, Vienna, Austria). The packages and complete version information are provided in Supplementary S26.

## 3. Results

### 3.1. Participant Characteristics

Baseline clinical characteristics of the 21 participants are summarized in Table 1. Metabolomic and plasma glucose analyses included 20 participants. Among these, nine used an automated insulin delivery system, and 11 used a non-automated insulin pump with continuous glucose monitoring.

### 3.2. Glycemic Responses to Exercise

Plasma glucose concentrations remained stable across the exercise protocol (repeated-measures ANOVA: F(3, 57) = 0.47, *p* = 0.705), with no significant differences between rest (8.65 ± 2.77 mmol/L) and the AT (8.63 ± 2.55 mmol/L), peak exercise (8.72 ± 2.60 mmol/L), or recovery (8.81 ± 2.61 mmol/L; all Holm-adjusted *p* = 1.000; Supplementary Tables S3 and S4).

### 3.3. Intensity-Dependent Changes in the Metabolomic Responses to Exercise

For each exercise workload, descriptive relative percentage changes in mean metabolite abundance from rest are described below, and the corresponding values are provided in Supplementary Table S1B.

#### 3.3.1. AT

At the AT, two metabolites with BH-FDR-significant Model 1 effects increased relative to rest: L-(+)-lactic acid (+21%) and 2-hydroxyisobutyric acid (+19%).

#### 3.3.2. Peak

Marked changes were observed at peak exercise. Increases were observed in metabolites related to glycolysis and TCA cycle, including malic acid (+124%), succinic acid (+90%), L-(+)-lactic acid (+79%), 2-hydroxyisobutyric acid (+53%), α-ketoglutarate (+48%), fumaric acid (+42%), and pyruvic acid (+27%), together with a pronounced increase in the purine catabolite hypoxanthine (+107%). In parallel, several fatty acids and other metabolites decreased, including oleic acid (−47%), oxalic acid (−45%), glutamic acid (−34%), stearic acid (−32%), 2-hydroxybutyric acid (−28%), palmitic acid (−25%), dihydrouracil (−19%), and glycerol (−3%).

#### 3.3.3. Recovery

During recovery, many of these changes persisted or intensified. The largest increases relative to rest were in taurine (+312%), malic acid (+244%), hypoxanthine (+222%), α-ketoglutarate (+144%), and fumaric acid (+112%), followed by L-(+)-lactic acid (+80%), succinic acid (+68%), alanine (+66%), pyruvic acid (+61%), 2-hydroxyisobutyric acid (+55%), and ketovaline (+53%). More modest increases were seen for dodecanoic acid (+32%), (±)-α-tocopherol (+32%), oleic acid (+26%), and cholesterol (+13%). Conversely, oxalic acid (−48%), L-(−)-sorbose (−46%), L-(+)-erythrulose (−43%), aminomalonic acid (−37%), dihydrouracil (−25%), and glycerol (−4%) remained reduced.

### 3.4. Determinants of the Metabolomic Responses to Exercise

Model 1 identified significant metabolite changes across the exercise stages. At the AT, only two metabolites were significantly increased relative to rest.

At peak exercise, 16 metabolites were significantly altered. The greatest increases were observed for malic acid (β = 1.02, 95% CI 0.75–1.29, *q* < 0.001), and L-(+)-lactic acid (β = 0.86, 95% CI 0.72–1.00, *q* < 0.001), whereas oxalic acid showed the largest decrease (β = −0.89, 95% CI −1.31 to −0.47, *q* < 0.01). The remaining TCA-cycle intermediates were also significantly increased (succinic acid β = 0.85, 95% CI 0.72–0.98; α-ketoglutarate β = 0.65, 95% CI 0.33–0.98; fumaric acid β = 0.35, 95% CI 0.19–0.51; all *q* ≤ 0.003).

During recovery, 21 metabolites were significantly altered, including increases in taurine (β = 2.64, 95% CI 1.18–4.10, *q* < 0.01) and malic acid (β = 1.62, 95% CI 1.35–1.89, *q* < 0.001), while oxalic acid showed the largest decrease (β = −1.09, 95% CI −1.51 to −0.66, *q* < 0.001). Selected significant results from Models 1 and 5 are provided in Table 2, the complete set of 39 BH-FDR-significant Model 1 workload–metabolite associations is provided in Supplementary Table S2A.

Workload-specific patterns among metabolites with at least one BH-FDR-significant Model 1 workload coefficient are shown in Supplementary Figure S2, while the corresponding Model 1 coefficients and 95% confidence intervals are shown in Supplementary Figure S3. Volcano plots for AT, peak exercise, and recovery are presented in Supplementary Figures S4–S6, respectively.

**Figure 1:**
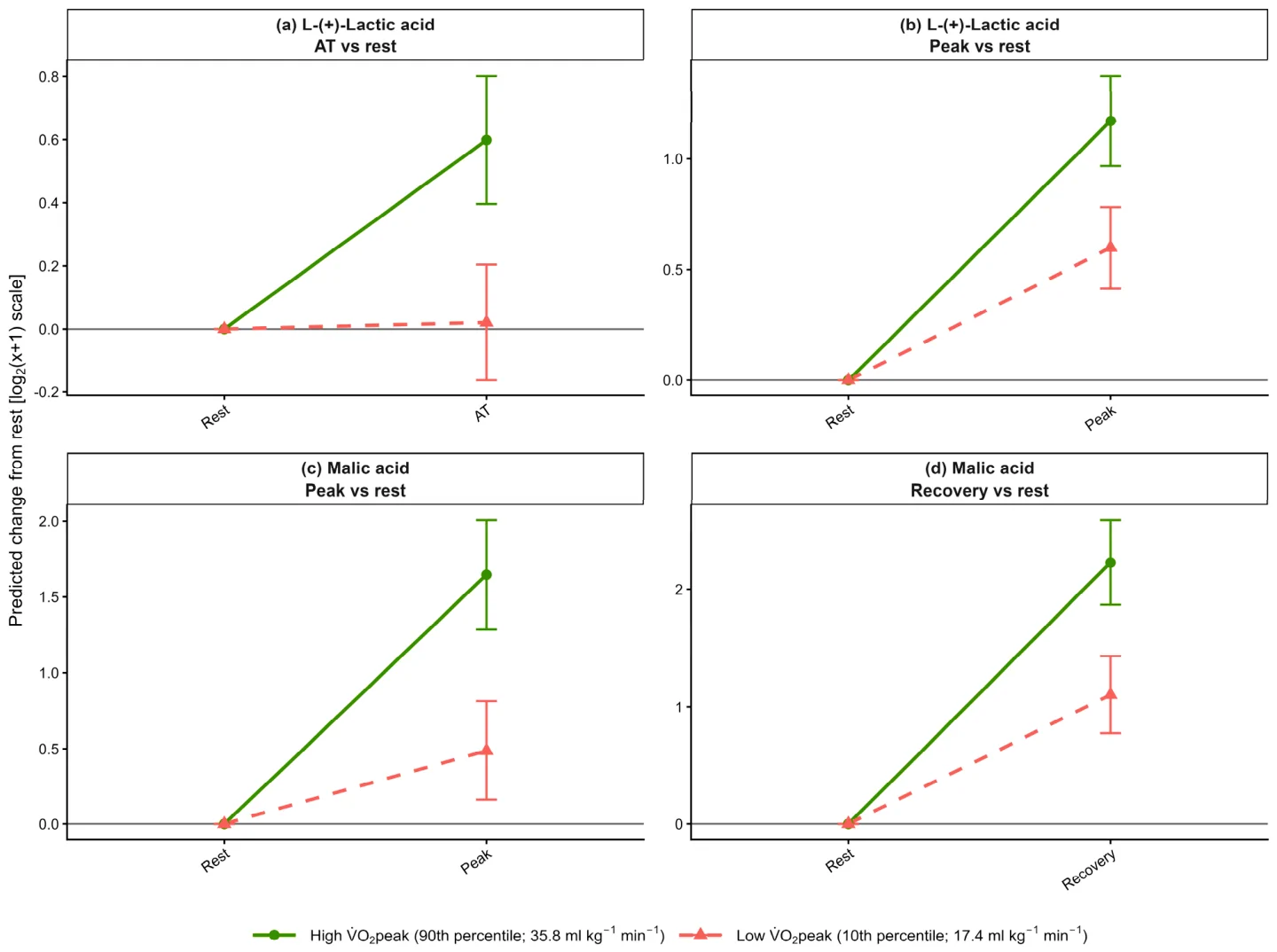
Model-predicted exercise-induced metabolite responses according to cardiorespiratory fitness (n = 20). Panels show the four BH-FDR-significant workload × V̇O_2peak_ interactions from Model 5: (**a**) L-(+)-lactic acid at the anaerobic threshold (β = 0.032, *q* = 0.040); (**b**) L-(+)-lactic acid at peak exercise (β = 0.031, *q* = 0.024); (**c**) malic acid at peak exercise (β = 0.063, *q* = 0.010); and (**d**) malic acid during recovery (β = 0.061, *q* = 0.015). Solid green lines with circles represent high V̇O_2peak_ (90th percentile; 35.8 mL kg^−1^ min^−1^), whereas dashed coral lines with triangles represent low V̇O_2peak_ (10th percentile; 17.4 mL kg^−1^ min^−1^). Points represent fixed-effect workload-minus-rest contrasts on the log_2_(x + 1)-transformed abundance scale and error bars represent 95% Wald confidence intervals. Continuous covariates were held at their mean values and sex at the female reference category. Corresponding predicted values are provided in Supplementary Table S2B. AT, anaerobic threshold; BH-FDR, Benjamini–Hochberg false discovery rate; CRF, cardiorespiratory fitness.

In the exploratory Model 5 analysis, BH-FDR significant workload × V̇O_2peak_ interactions were identified for L-(+)-lactic acid and malic acid. Higher V̇O_2peak_ was associated with greater increases in L-(+)-lactic acid at the AT (β = 0.032, 95% CI 0.015–0.048, *q* = 0.040) and peak exercise (β = 0.031, 95% CI 0.015–0.047, *q* = 0.024). Significant interactions were also observed for malic acid at peak exercise (β = 0.063, 95% CI 0.034–0.092, *q* = 0.010) and during recovery (β = 0.061, 95% CI 0.032–0.091, *q* = 0.015). These interactions are illustrated by model-predicted metabolite trajectories in Figure 1, with the corresponding predicted values reported in Supplementary Table S2B.

Unadjusted workload associations in Model 0 were consistent with those in the adjusted Model 1, identifying the same 39 significant workload-specific associations. The exploratory Models 2–4 provided no evidence of significant BH-FDR workload × HbA1c or workload × resting glucose interactions, and no significant independent associations between V̇O_2peak_ and metabolite abundance. Main effects of glycemia were, however, observed for a small number of metabolites: resting glucose was associated with five metabolites in Model 3, and HbA1c with 2-hydroxybutyric acid across Models 1, 2, 4 and 5 and with hypoxanthine in Models 4 and 5 (Supplementary Table S2A).

### 3.5. Correlations Between Metabolic Responses and Clinical Characteristics

**Figure 2:**
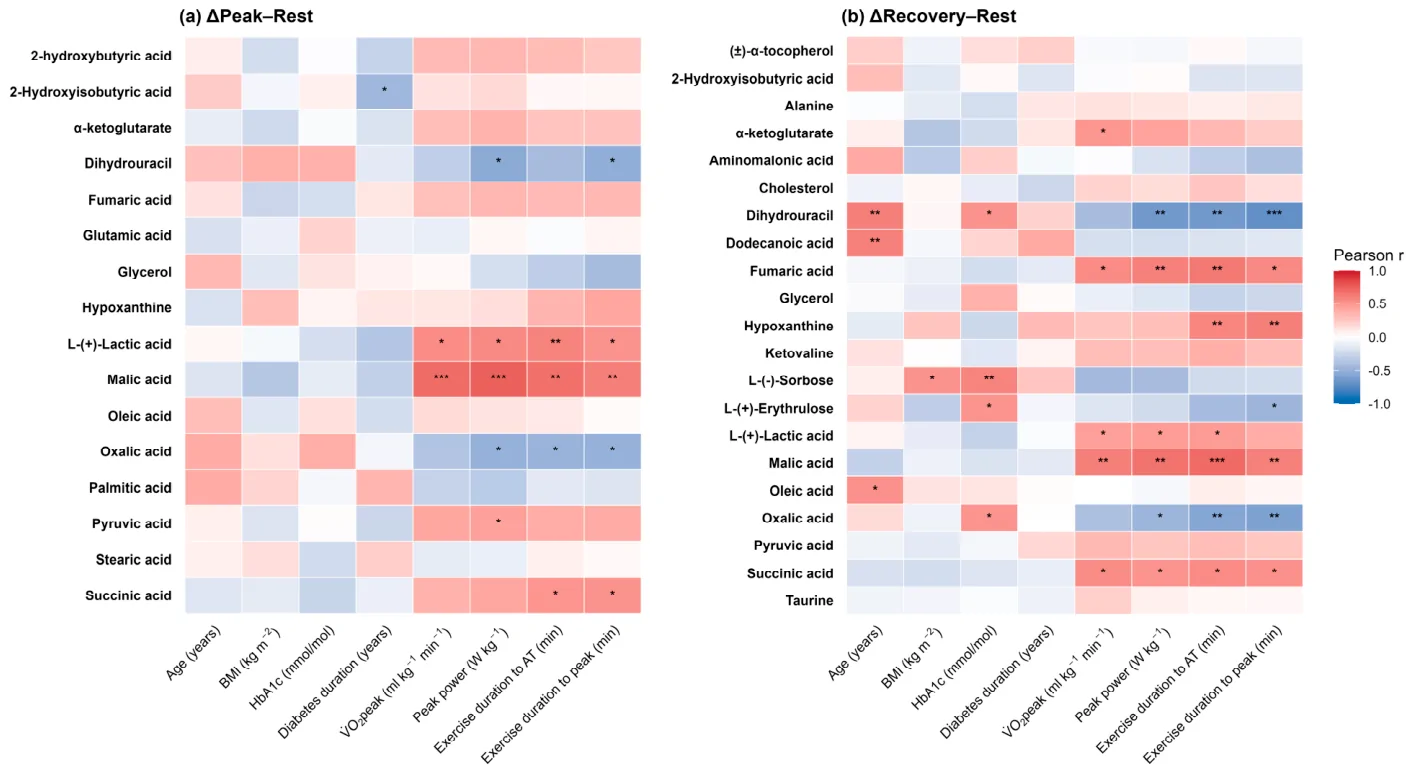
Exploratory correlations between exercise-induced metabolite changes and clinical and exercise characteristics (n = 20): (**a**) peak exercise minus rest and (**b**) recovery minus rest. For each contrast, the analysis was restricted to metabolites with a BH-FDR-significant Model 1 coefficient for that same contrast. Metabolite changes were calculated on the log_2_(x + 1)-transformed abundance scale. Colors represent Pearson correlation coefficients. * *p* < 0.05, ** *p* < 0.01 and *** *p* < 0.001, based on unadjusted two-sided *p* values. AT, anaerobic threshold; BH-FDR, Benjamini–Hochberg false discovery rate; V̇O_2peak_, peak oxygen uptake.

Correlation heatmaps for ΔPeak–Rest and ΔRecovery–Rest are presented in Figure 2, whereas correlations for ΔAT–Rest are shown in Supplementary Figure S7. Complete correlation coefficients are provided in Supplementary Table S5. These analyses were exploratory and hypothesis-generating; *p*-values were two-sided and were not adjusted for multiple testing.

At ΔPeak–Rest (Figure 2a), changes in malic acid demonstrated strong positive correlations with peak power (r = 0.74, *p* < 0.001) and V̇O_2peak_ (r = 0.69, *p* < 0.001). In addition, L-(+)-lactic acid showed positive correlations with V̇O_2peak_ (r = 0.54, *p* = 0.014) and peak power (r = 0.56, *p* = 0.011).

At ΔRecovery–Rest (Figure 2b), a similar pattern was observed. Malic acid continued to correlate strongly with V̇O_2peak_ (r = 0.60, *p* = 0.005) and peak power (r = 0.66, *p* = 0.002). Positive associations were also observed for L-(+)-lactic acid with V̇O_2peak_ (r = 0.45, *p* = 0.047) and peak power (r = 0.46, *p* = 0.040).

Malic acid was also positively correlated with time to the AT at both ΔPeak–Rest (r = 0.67, *p* = 0.001) and ΔRecovery–Rest (r = 0.69, *p* < 0.001). At ΔPeak–Rest, L-(+)-lactic acid was also positively correlated with time to AT (r = 0.59, *p* = 0.007).

### 3.6. Metabolic-Category Over-Representation Analysis

**Figure 3:**
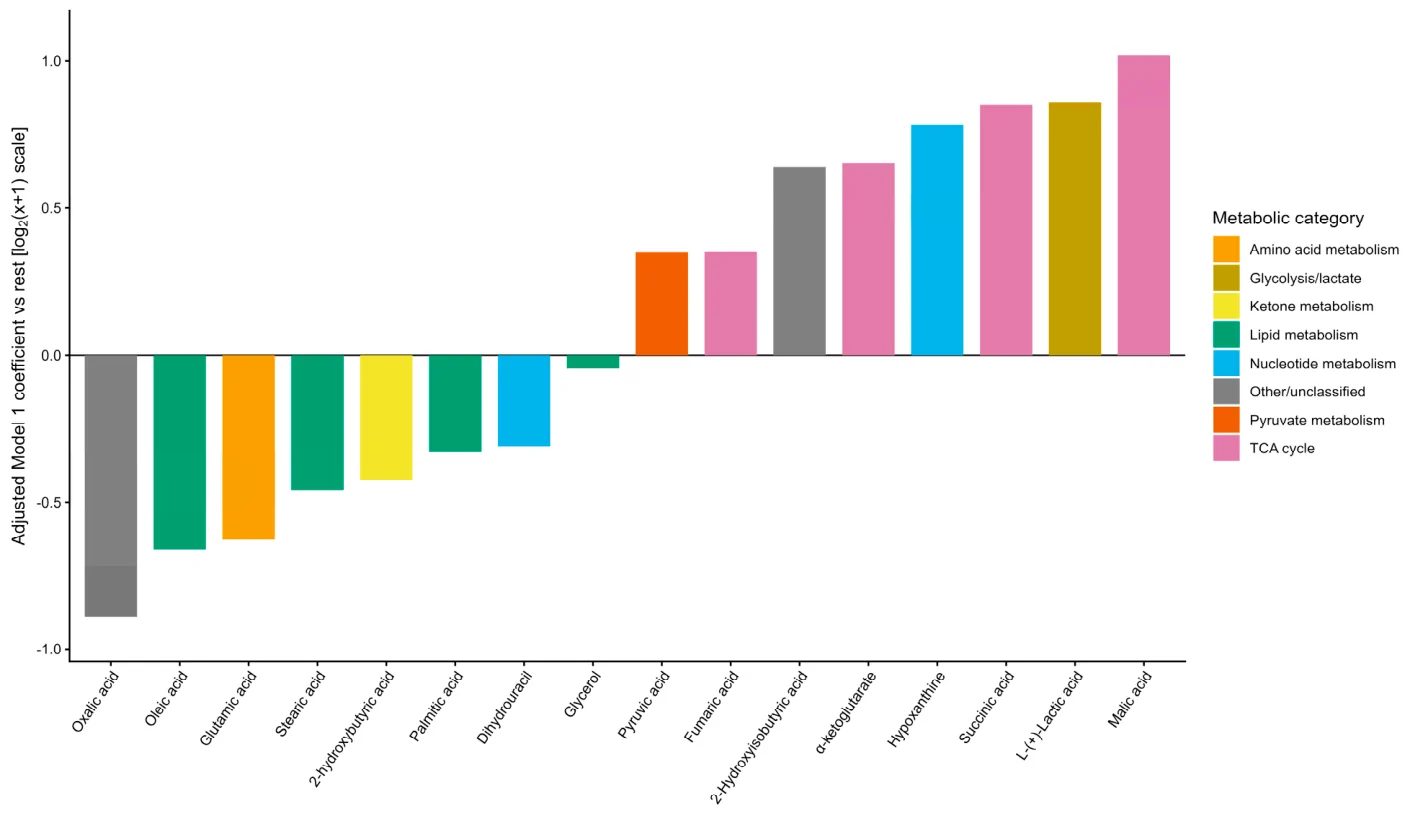
Broad metabolic-category classification of metabolites with BH-FDR-significant Model 1 coefficients for peak exercise vs. rest (*q* < 0.05; n = 20). Bars represent adjusted coefficients on the log_2_(x + 1)-transformed abundance scale; positive and negative coefficients indicate higher and lower abundance at peak exercise, respectively. Colors indicate broad metabolic categories assigned using KEGG annotation where available, supplemented by predefined biochemical classification; metabolites without a specific assignment were classified as other/unclassified. BH-FDR, Benjamini–Hochberg false discovery rate; KEGG, Kyoto Encyclopedia of Genes and Genomes; TCA, tricarboxylic acid.

Figure 3 and Supplementary Figure S8 show the broad metabolic-category classification of metabolites that changed significantly at peak and recovery, respectively. The corresponding classification of the two metabolites significantly increased at the AT is shown in Supplementary Figure S9. Relative percentage changes are provided in Supplementary Table S1B, while the corresponding LMM results are provided in Table 2 and Supplementary Table S2A.

At peak exercise (Figure 3), the largest increases included TCA cycle intermediates such as malic acid, α-ketoglutarate, succinic acid, and fumaric acid, together with increases in L-(+)-lactic acid and pyruvic acid. Within the nucleotide metabolism category, hypoxanthine increased, whereas dihydrouracil decreased. Several lipid-related metabolites, including palmitic acid, stearic acid, and oleic acid, also decreased, together with glutamic acid and oxalic acid.

During recovery (Supplementary Figure S8), substantial changes in energy-related metabolites persisted. Taurine exhibited the largest increase, followed by sustained elevations in hypoxanthine; the TCA cycle intermediates malic acid, α-ketoglutarate, and fumaric acid; the glycolysis-related metabolites L-(+)-lactic acid and pyruvic acid; and the amino acid alanine. Reductions persisted in oxalic acid, dihydrouracil, and L-(+)-erythrulose.

Metabolic-category over-representation analysis showed significant over-representation of the TCA cycle at peak exercise (*q* = 0.0027) and during recovery (*q* = 0.0085). The nucleotide metabolism was also significantly over-represented at peak exercise (fold enrichment = 10.25, *p* = 0.009, *q* = 0.0404). This finding was based on significant changes in both measured metabolites assigned to this category, hypoxanthine and dihydrouracil. Because the category contained only these two metabolites, the result should be interpreted as over-representation within the measured metabolite panel and not as evidence of changes across the complete nucleotide-metabolism pathway. The pyruvate-metabolism category, which contained only one measured metabolite, was not significantly over-represented at peak exercise or during recovery. No metabolic category was significantly over-represented at the AT (Supplementary Table S6).

## 4. Discussion

This study used targeted and untargeted metabolomics to characterize the plasma metabolome throughout maximal exercise testing and investigate the metabolic signatures associated with CRF in adults with type 1 diabetes. Metabolic perturbations were comparatively limited at the anaerobic threshold but became widespread at peak exercise and persisted into early recovery. Furthermore, higher V̇O_2peak_ was associated with greater exercise-induced changes in specific glycolytic and TCA-cycle metabolites. Collectively, these exploratory findings indicate intensity-dependent changes in the circulating metabolome during exercise and suggest preliminary associations between CRF and the magnitude of specific metabolic responses to maximal exercise.

Our graded exercise model enabled us to comprehensively capture the dynamic evolution of energy metabolism across progressively increasing physiological demand. At the anaerobic threshold, changes were comparatively limited, with significant increases observed only in L-(+)-lactic acid and 2-hydroxyisobutyric acid and no metabolic-category over-representation. In contrast, maximal exercise elicited coordinated increases in the glycolysis-related metabolites L-(+)-lactic acid and pyruvic acid and the TCA-cycle intermediates succinic acid, malic acid, fumaric acid and α-ketoglutarate. Over-representation analysis indicated that these changes were pathway-coordinated rather than isolated, with significant enrichment of the TCA cycle and nucleotide metabolism at peak exercise. Hypoxanthine also increased significantly, consistent with increased purine degradation under high energetic demand. These responses were accompanied by reductions in the long-chain fatty acids oleic, palmitic and stearic acids, consistent with an intensity-dependent change in substrate mobilization and utilization [12,28].

Exercise cessation did not result in an immediate return of the metabolome toward baseline. During early recovery, glycolysis-related and TCA-cycle metabolites remained markedly elevated, with several demonstrating numerically larger differences from rest than at peak exercise. Hypoxanthine was 222% above rest during recovery, supporting continued purine degradation after the cessation of muscular work [29]. Recovery was also characterized by increases in several amino-acid- and lipid-related metabolites, including taurine, alanine, ketovaline and oleic acid. Although the tissue origins and physiological significance of these circulating responses cannot be determined from the present data, their magnitude and diversity indicate that early recovery represents an active phase of substrate redistribution rather than an immediate return to resting homeostasis.

These intensity-dependent changes are consistent with the close coupling between ATP production (via glycolysis and the TCA cycle) and ATP turnover (via purine degradation), providing novel insight into the metabolic demands underpinning exercise capacity in type 1 diabetes. From a methodological perspective, the rapid evolution of these responses also highlights the importance of serial sampling when characterizing the exercise metabolome.

The application of metabolomics around exercise in type 1 diabetes remains limited and has largely been confined to fixed exercise-intensity protocols with metabolomic profiling performed at rest or before and after exercise [16,17,18,19]. Using a high-intensity cycling protocol (30 min at ~80% V̇O_2max_), Brugnara et al. reported attenuated increases in TCA-cycle intermediates together with blunted lipid and amino-acid metabolic responses in adults with type 1 diabetes compared with healthy controls [16]. Bally et al. subsequently demonstrated that continuous and intermittent exercise elicited distinct perturbations in purine and acylcarnitine metabolism, highlighting the sensitivity of the exercise metabolome to exercise modality [17]. More recently, Taylor et al. showed that the resting serum malic-to-pyruvate ratio could distinguish individuals with high and low CRF and that metabolomic responses to moderate-intensity continuous exercise were influenced by residual β-cell function [19]. Consistent with the sensitivity of the exercise metabolome to energetic stress, we previously demonstrated that a sustained bout of submaximal cycling (45 min at 60% V̇O_2peak_) performed during hypoglycemia potentiated perturbations in purine metabolism and the purine salvage pathway [18]. In contrast, the present study employed an exercise protocol designed to minimize disturbances in ambient glucose concentrations, reducing the likelihood that the observed metabolomic changes were primarily driven by acute glycemic fluctuations. Collectively, these studies indicate that the exercise metabolome is shaped by multiple interacting factors, including disease status, residual β-cell function, exercise modality, glycemic state and exercise intensity.

The exercise-induced metabolic responses observed in the present study broadly align with those reported in the general population, encompassing changes in circulating glycolysis-related metabolites, TCA-cycle intermediates and purine metabolites [30,31]. In keeping with previous studies in healthy individuals [32,33,34,35], adults with type 1 diabetes with higher CRF demonstrated greater exercise-induced changes in selected metabolites involved in central energy metabolism, including L-(+)-lactic acid and malic acid. As fitter individuals can sustain higher external workloads, these responses may partly reflect the greater bioenergetic demands imposed during maximal exercise [35,36,37]. Due to their interconnectedness, the effects of internal versus external ‘work’ cannot be fully disentangled in this design.

The exploratory adjusted associations between CRF and exercise-induced changes in glycolytic and TCA-cycle metabolites may be consistent with fitter individuals exhibiting a greater capacity to mount the metabolic response required to sustain maximal exercise. However, these associations may be unstable because of the small sample size and should not be interpreted as evidence of a causal or independent physiological mechanism. Furthermore, circulating metabolite concentrations do not directly measure tissue-specific metabolic flux. Confirmation in larger cohorts using direct assessments of metabolic flux and mitochondrial function is therefore required.

In bivariate analyses, exercise-induced changes in several glycolytic and TCA-cycle metabolites demonstrated more consistent relationships with CRF, peak power and exercise duration than with HbA1c. Isolated main effects of glycemia were nonetheless observed, with resting glucose positively associated with five metabolites and HbA1c with 2-hydroxybutyric acid and hypoxanthine. These exploratory signals were mainly observed for hydroxybutyrate and sugar-related metabolites rather than the glycolytic and TCA-cycle intermediates that tracked CRF. This pattern may be consistent with glycemia influencing resting substrate availability; however, the small sample size does not allow us to determine whether glycemic control influences the magnitude of the exercise response. From a clinical perspective, these findings may suggest that the metabolic response to exercise may provide information about functional health that is not captured by conventional measures of glycemic control alone.

Although the clinical utility of implementing metabolomics in routine care for individuals with type 1 diabetes remains to be established, growing evidence suggests that CRF-associated metabolomic signatures may have broader prognostic relevance. Indeed, a recent UK Biobank study demonstrated that metabolomic signatures associated with CRF were independently associated with substantially lower risks of all-cause mortality, cardiovascular disease, type 2 diabetes and colorectal cancer [20].

### 4.1. Strengths, Limitations and Perspectives for Future Research

#### Strengths

A major strength of this study was the integration of targeted and untargeted metabolomics with serial blood sampling throughout a graded maximal exercise test, enabling comprehensive characterization of the temporal evolution of the exercise metabolome across progressively increasing physiological demand. Unlike previous studies employing fixed exercise protocols or pre- and post-exercise sampling alone, our approach captured the dynamic metabolic transitions accompanying increasing exercise intensity and recovery. Additionally, participants spanned a broad range of age, diabetes duration and CRF, providing substantial interindividual variability in the characteristics relevant to the present analyses.

The repeated -measures design allowed each participant to serve as their own control when estimating exercise-stage changes, while the participant-specific random intercept accounted for the correlation among repeated measurements. The application of BH-FDR correction across metabolites within each model term and metabolic-category enrichment analyses supported the exploratory biological interpretation of the results. These methodological features, however, do not overcome the limitations associated with the small number of independent participants.

### 4.2. Limitations

The principal limitations of this exploratory secondary analysis are the small sample size and absence of a healthy control group, the latter precluding determination of whether the observed exercise-induced metabolic responses are specific to type 1 diabetes or reflect physiological responses to exercise more generally. The blood sample selected to represent AT did not always coincide exactly with the modeled AT, which may have attenuated the metabolite responses observed at this workload. In addition, quantile normalization was used to harmonize abundance distributions across samples; however, this distribution-based procedure may have attenuated genuine global metabolic changes induced by exercise, and no sensitivity analysis using an alternative normalization approach was performed. Although the timing of food intake was standardized, meal composition was not standardized and may therefore have contributed to residual metabolic variation. Despite implementing a highly standardized pre-testing protocol that controlled the timing of dietary intake and insulin administration as well as exposure to recent hypoglycemia and physical activity, residual confounding by these factors could not be fully excluded. Furthermore, circulating insulin concentrations were not measured, and C-peptide concentrations were available in only 13 participants (62%), with very low concentrations in the majority, limiting our ability to assess the influence of exogenous and residual endogenous insulin exposure on the observed exercise-induced metabolomic responses. No separate a priori sample-size calculation was performed because the available sample was fixed by the two parent trials. Although BH-FDR correction was applied across metabolites within each model term, this addresses multiple testing but does not eliminate the imprecision or potential model instability associated with the small sample.

### 4.3. Future Research

The results of this study should be considered as hypothesis generating, and their potential clinical relevance requires confirmation in larger, independent populations across different exercise stimuli. Integrating serial exercise metabolomics with direct assessments of skeletal muscle mitochondrial function, in vivo metabolic flux, purine turnover, oxidative stress and antioxidant defences, alongside longitudinal changes in CRF following training would further elucidate the biological mechanisms underlying exercise capacity and recovery. Such work may also help determine whether exercise-induced metabolomic signatures have potential utility as biomarkers of CRF and exercise capacity in adults with type 1 diabetes.

## 5. Conclusions

In this exploratory pooled secondary analysis exercise intensity was associated with changes in glycolysis-related, TCA-cycle and purine metabolites which were most pronounced at peak exercise and persisting into early recovery. Furthermore, preliminary associations were also observed between V̇O_2peak_ and exercise-induced L-(+)-lactic acid and malic acid. These findings may suggest that exercise metabolomics provides physiological information beyond conventional measures of glycemic control and may offer complementary insight into the biological mechanisms underpinning exercise capacity in adults with type 1 diabetes.

## Figures and Tables

**Table 1:** Baseline clinical characteristics of the study participants (n = 21).

Variable	n (%)	Median (IQR)	Mean ± SD	Range
**Demographics**				
Age (years)	21	52 (43–60)	51.6 ± 13.2	26–72
Age at diabetes onset (years)	21	19 (11–30)	21.2 ± 13.3	1–43
Diabetes duration (years)	21	30 (20–40)	30.4 ± 13.0	12–57
BMI (kg/m^2^)	21	27.0 (25.4–29.6)	27.6 ± 3.8	21.0–33.8
**Sex**	21			
Females	14 (66.7)			
Males	7 (33.3)			
**Cardiovascular**				
SBP (mmHg)	21	135 (125–141)	132.8 ± 10.2	112–149
DBP (mmHg)	21	83 (78–88)	81.1 ± 10.3	55–94
Pulse (bpm)	21	65 (62–82)	69.4 ± 12.1	49–88
**Glycemic control**				
HbA1c (mmol/mol)	21	67 (62–72)	67.5 ± 7.0	59–86
HbA1c (%)	21	8.3 (7.8–8.7)	8.3 ± 0.6	7.5–10.0
**Lipids**				
Total cholesterol (mmol/L)	21	4.6 (4.3–4.9)	4.63 ± 0.62	3.4–5.8
LDL-cholesterol (mmol/L)	21	2.3 (2.0–2.4)	2.25 ± 0.49	1.1–3.5
HDL-cholesterol (mmol/L)	21	1.87 (1.48–2.25)	1.92 ± 0.49	1.17–3.11
Triglycerides (mmol/L)	21	0.94 (0.70–1.36)	1.01 ± 0.37	0.47–1.61
**Exercise testing**				
Peak power (W)	21	185 (155–240)	193.8 ± 63.9	95–320
Peak power (W/kg)	21	2.43 (1.81–3.01)	2.44 ± 0.72	1.42–4.10
V̇O_2_peak (ml min^−1^ kg^−1^)	21	25.2 (19.2–28.0)	25.8 ± 7.7	12.1–45.0
V̇O_2_ at AT (ml min^−1^ kg^−1^)	21	20.7 (17.0–21.6)	20.8 ± 6.1	12.7–37.1
Time to AT (min)	21	11.6 (9.8–13.5)	11.5 ± 2.3	7.0–15.3
Time to peak (min)	21	16.5 (14.0–18.0)	15.8 ± 3.2	10.4–22.8

**Footnote:** Data are presented as n (%), median (IQR), mean ± SD and range. HbA1c is reported in IFCC (mmol/mol) and NGSP (%) units; the percentage values were converted using NGSP (%) = 0.09148 × IFCC (mmol/mol) + 2.152. AT, anaerobic threshold; BMI, body mass index; DBP, diastolic blood pressure; HDL, high-density lipoprotein; IQR, interquartile range; LDL, low-density lipoprotein; SBP, systolic blood pressure; V̇O_2peak_, peak oxygen uptake.

**Table 2:** Selected significant metabolite associations identified by linear mixed-effects Models 1 and 5.

Model	Metabolite	Model Term	Estimate	95% CI	*p* Value	*q* Value	Significance
M1	2-Hydroxyisobutyric acid	AT	0.281	[0.176, 0.387]	<0.0001	0.0002	***
M1	2-Hydroxyisobutyric acid	Peak	0.637	[0.531, 0.742]	<0.0001	<0.0001	***
M1	2-Hydroxyisobutyric acid	Recovery	0.66	[0.555, 0.766]	<0.0001	<0.0001	***
M1	Hypoxanthine	Peak	0.783	[0.289, 1.278]	0.0024	0.0289	*
M1	Hypoxanthine	Recovery	1.663	[1.168, 2.157]	<0.0001	<0.0001	***
M1	L-(+)-Lactic acid	AT	0.286	[0.145, 0.426]	0.0001	0.0099	**
M1	L-(+)-Lactic acid	Peak	0.86	[0.719, 1.000]	<0.0001	<0.0001	***
M1	L-(+)-Lactic acid	Recovery	0.874	[0.734, 1.015]	<0.0001	<0.0001	***
M1	Malic acid	Peak	1.019	[0.749, 1.288]	<0.0001	<0.0001	***
M1	Malic acid	Recovery	1.619	[1.350, 1.889]	<0.0001	<0.0001	***
M1	Oleic acid	Peak	−0.663	[−1.017, −0.309]	0.0004	0.0074	**
M1	Oleic acid	Recovery	0.51	[0.156, 0.864]	0.0055	0.0430	*
M1	Oxalic acid	Peak	−0.891	[−1.315, −0.466]	<0.0001	0.0020	**
M1	Oxalic acid	Recovery	−1.085	[−1.510, −0.661]	<0.0001	<0.0001	***
M1	Pyruvic acid	Peak	0.348	[0.156, 0.540]	0.0006	0.0097	**
M1	Pyruvic acid	Recovery	0.702	[0.510, 0.893]	<0.0001	<0.0001	***
M1	Succinic acid	Peak	0.852	[0.723, 0.981]	<0.0001	<0.0001	***
M1	Succinic acid	Recovery	0.693	[0.564, 0.822]	<0.0001	<0.0001	***
M1	Fumaric acid	Peak	0.349	[0.186, 0.512]	<0.0001	0.0020	**
M1	Fumaric acid	Recovery	0.757	[0.594, 0.920]	<0.0001	<0.0001	***
M1	alpha-ketoglutarate	Peak	0.653	[0.331, 0.976]	0.0001	0.0030	**
M1	alpha-ketoglutarate	Recovery	1.324	[1.002, 1.646]	<0.0001	<0.0001	***
M1	Taurine	Recovery	2.64	[1.178, 4.102]	0.0006	0.0068	**
M5	L-(+)-Lactic acid	AT × V̇O_2_peak interaction	0.032	[0.015, 0.048]	0.0002	0.0403	*
M5	L-(+)-Lactic acid	Peak × V̇O_2_peak interaction	0.031	[0.015, 0.047]	0.0003	0.0235	*
M5	Malic acid	Peak × V̇O_2_peak interaction	0.063	[0.034, 0.092]	<0.0001	0.0095	**
M5	Malic acid	Recovery × V̇O_2_peak interaction	0.061	[0.032, 0.091]	<0.0001	0.0148	*

**Footnote:** Model 1 included exercise workload, age, sex, HbA1c and diabetes duration. Model 5 additionally included mean-centered V̇O_2peak_ and the workload × V̇O_2peak_ interaction. Model 1 estimates represent adjusted differences in log_2_(x + 1)-transformed metabolite abundance relative to rest. Model 5 interaction estimates represent the change in the workload effect per 1 mL min^−1^ kg^−1^ higher V̇O_2peak_. Benjamini–Hochberg false discovery rate correction was applied separately within each model term. Metabolomic analyses included 20 participants. * *q* < 0.05, ** *q* < 0.01 and *** *q* < 0.001. AT, anaerobic threshold; CI, confidence interval; V̇O_2peak_, peak oxygen uptake. The complete set of 39 BH-FDR-significant Model 1 workload–metabolite associations is provided in Supplementary Table S2A.

## Data Availability

The pseudonymized participant-level metabolomics and clinical data underlying this study are not publicly available because unrestricted public disclosure is not covered by the applicable participant-consent, institutional data-governance and data-protection arrangements. Data may be made available to qualified researchers upon reasonable request to the corresponding author, subject to approval by the responsible data controller at Steno Diabetes Center Copenhagen, fulfillment of applicable ethical and legal requirements, and establishment of an appropriate institutional data-sharing or data-processing agreement. Aggregate results supporting the conclusions of this study are provided in the article and its Supplementary Materials.

## Supplementary Materials

The following supporting information can be downloaded at: https://www.mdpi.com/article/10.3390/metabo16090687/s1, Supplementary Methods; Supplementary Table S1A: Descriptive statistics for raw targeted and untargeted metabolomic data before log transformation; Supplementary Table S1B: Relative percentage changes in metabolite abundance between exercise stages; Supplementary Table S2A: Significant fixed effects identified by linear mixed-effects Models 0–5 following Benjamini–Hochberg false discovery rate correction; Supplementary Table S2B: Model-predicted workload-minus-rest metabolite responses at low (10th percentile) and high (90th percentile) cardiorespiratory fitness; Supplementary Table S3: Descriptive statistics of plasma glucose concentrations across exercise workloads; Supplementary Table S4: Holm-adjusted pairwise comparisons of plasma glucose concentrations across exercise workloads; Supplementary Table S5: Pearson correlations between workload-specific metabolite changes and clinical characteristics, cardiorespiratory fitness, and glycemic control; Supplementary Table S6: Over-representation analysis of broad metabolic categories among metabolites with BH-FDR-significant Model 1 workload effects; Supplementary Figure S1. Overview of the graded incremental cardiopulmonary exercise test and blood-sampling protocol. Supplementary Figure S2: Heatmap of the 25 unique metabolites with at least one BH-FDR-significant Model 1 workload coefficient; Supplementary Figure S3: Forest plot of BH-FDR-significant adjusted Model 1 workload coefficients for the anaerobic threshold, peak exercise, and recovery versus rest; Supplementary Figure S4: Volcano plot of adjusted Model 1 coefficients for the anaerobic threshold versus rest; Supplementary Figure S5: Volcano plot of adjusted Model 1 coefficients for peak exercise versus rest; Supplementary Figure S6: Volcano plot of adjusted Model 1 coefficients for recovery versus rest; Supplementary Figure S7: Exploratory Pearson correlations between metabolite changes from rest to the anaerobic threshold and clinical and exercise characteristics; Supplementary Figure S8: Broad metabolic-category classification of metabolites with BH-FDR-significant Model 1 coefficients for recovery versus rest, Supplementary Figure S9: Broad metabolic-category classification of metabolites with BH-FDR-significant Model 1 coefficients for the anaerobic threshold versus rest.

## Abbreviations

The following abbreviations are used in this manuscript:

AID	Automated insulin delivery
AT	Anaerobic threshold
ATP	Adenosine triphosphate
BH-FDR	Benjamini–Hochberg false discovery rate
BMI	Body mass index
CI	Confidence interval
CPET	Cardiopulmonary exercise testing
CRF	Cardiorespiratory fitness
GC–MS	Gas chromatography–mass spectrometry
HbA1c	Glycated hemoglobin
IQR	Interquartile range
KEGG	Kyoto Encyclopedia of Genes and Genomes
LMM	Linear mixed-effects model
QC	Quality control
RSD	Relative standard deviation
SD	Standard deviation
T1D	Type 1 diabetes
TCA	Tricarboxylic acid cycle
V̇O_2peak_	Peak oxygen uptake
