# Supplementary material for: Exercise Metabolomics Reveals Intensity-Dependent Metabolic Responses Associated with Cardiorespiratory Fitness in Adults with Type 1 Diabetes
Source: Metabolites. 2026 Sep 18;16(9):687. doi: 10.3390/metabo16090687 (PMC13608913; doi:10.3390/metabo16090687)
Supplement: Supplementary file 1 [file metabolites-16-00687-s001.zip › metabolites-4505348-Supplementary-Materials_section.pdf]

Exercise Metabolomics Reveals Intensity-Dependent Metabolic Responses Associated with Cardiorespiratory Fitness in Adults with Type 1 Diabetes

Olivia M. McCarthy 1,2*, Clara Solà 1, Chloe Nicholas 1,2, Sandra Tawfik 1, Merete Bechmann Christensen 1, Signe Schmidt 1, Kirsten Nørgaard 1,3, Richard M. Bracken 2,4 and Marietta Kokla 1

Supplementary Materials

Supplementary Methods

Supplementary Section S1. Study design and source studies

The present study was a pooled secondary analysis of two investigator-initiated, randomized crossover clinical trials registered at ClinicalTrials.gov (NCT05133765 and NCT05134025) and sponsored by Steno Diabetes Center Copenhagen. Both trials used the same cardiopulmonary exercise-testing protocol during the screening visit.

Only data collected during the common screening protocol were included in the present analysis. Measurements and outcomes from the subsequent randomized intervention periods were not included. Accordingly, randomized treatment allocation was neither an exposure nor a comparator in this analysis.

The trials were conducted in accordance with the Declaration of Helsinki and approved by the National Research Ethics Committee of the Capital Region of Denmark (H-19029796). All participants received written and verbal information about the study procedures and provided written informed consent before participation.

Supplementary Section S2. Eligibility criteria

Participants were eligible if they were aged 18–75 years, had type 1 diabetes for ≥2 years, had HbA1c ≥58 mmol/mol (7.0%), had used an insulin pump for ≥12 months, had used a continuous or intermittently scanned glucose-monitoring system for ≥6 months and had used insulin aspart for ≥1 week.

The principal exclusion criteria were pregnancy or breastfeeding; use of glucose-lowering medications other than insulin; use of corticosteroids or other medications affecting glucose metabolism during the study period or within 30 days before enrolment; daily acetaminophen use; alcohol or drug misuse; and clinical conditions for which an HbA1c target below 53 mmol/mol (7.0%) was considered contraindicated. Eligibility was established within the parent trials before participants completed the screening cardiopulmonary exercise test.

Supplementary Section S3. Pre-exercise standardization

Participants were instructed to abstain from caffeine for 12 h and from alcohol and physical exercise for 24 h before laboratory testing. They were encouraged to avoid hypoglycemia, defined as a capillary fingertip glucose concentration ≤3.9 mmol/l, during the preceding 24 h. Participants maintained their usual insulin regimen and consumed a meal ≥2 h before the scheduled appointment. The composition of the pre-exercise meal was not standardized. Before exercise, participants were screened for absolute and relative contraindications to exercise stress testing according to American College of Sports Medicine guidelines. A peripheral intravenous cannula was inserted into the antecubital fossa for collection of venous blood throughout the exercise-testing visit.

Supplementary Section S4. Cardiopulmonary exercise-testing protocol

Participants performed a graded cardiopulmonary exercise test on a workload-controlled cycle ergometer using either a Corival ergometer (Lode, Groningen, the Netherlands) or a Monark LC4 ergometer (Vansbro, Sweden).

The protocol comprised:

- a 3 min seated rest phase;
- a 3 min warm-up at 20 W;
- an incremental exercise phase with 1 min increases in workload until volitional exhaustion;
- a 3 min active recovery phase at 20 W; and
- a 3 min passive recovery phase.

The incremental phase was intended to last approximately 10 min. The observed mean±SD duration of incremental exercise was 9.93±3.20 min.

Supplementary Section S5. Cardiopulmonary measurements

Breath-by-breath pulmonary gas-exchange data were recorded using a Vyntus ONE pulmonary gas analyzer (Vyaire Medical, Illinois, USA). Before each test, the analyzer was calibrated using ambient air and a certified calibration gas containing 15% oxygen and 5% carbon dioxide. Gas-exchange measurements were displayed under standardized temperature and pressure conditions for dry air.

Heart rate was recorded continuously using chest-belt telemetry (Polar Electro, Finland). Raw cardiopulmonary data were exported at 5 s intervals using SentrySuite software and averaged over consecutive 30 s periods for analysis.

The anaerobic threshold (AT) was determined using the Exercise Threshold App. Peak oxygen uptake ($\dot{V}O_{2peak}$) was defined as the highest oxygen-uptake value recorded during a 30 s interval before attainment of peak workload. $\dot{V}O_{2peak}$ was used rather than $\dot{V}O_{2max}$ because an oxygen-uptake plateau could not be identified in all participants.

Supplementary Section S6. Blood sampling and analytical time points

Venous blood was collected at rest before exercise, every 3 min during incremental exercise, at volitional exhaustion and during passive recovery 6 min after volitional exhaustion.

The four analytical workloads were defined as:

- Rest: the pre-exercise sample;
- Anaerobic threshold (AT): the exercise sample collected closest in time to the individually determined anaerobic threshold;
- Peak: the sample collected at volitional exhaustion; and
- Recovery: the sample collected 6 min after volitional exhaustion.

Among participants with both timing measurements available (n = 19), the modeled AT occurred at 11.47 ± 2.42 min, and the corresponding blood sample was collected at 11.21 ± 2.80 min. There was no evidence of asystematic difference between the the modeled AT and blood-sampling times(p = 0.181). At the individual level, the mean absolute timing difference was 43.2 ± 27.3 s (median, 45.0 s; IQR, 19.8–62.4 s). Fourteen of the 19 samples (73.7%) were collected within 60 s of the modeled AT, and all samples were collected within 90 s of the modeled AT.

Twenty-one participants completed the cardiopulmonary exercise test and were included in the clinical description. Metabolomic data were unavailable for one participant because of a technical failure during mass-spectrometric analysis. Plasma glucose measurements were also unavailable for this participant. Consequently, the metabolomic and plasma glucose analyses included the same 20 participants.

Supplementary Section S7. Plasma extraction and internal standards

A 30 µl plasma aliquot was extracted with methanol containing the following isotopically labeled internal standards:

- succinic acid-d4;
- glutamic acid-d5;
- valine-d8; and
- heptadecanoic acid-d33.

After vortex mixing and incubation on ice, samples were centrifuged to precipitate proteins. The resulting supernatants were transferred to clean analytical vials and dried under a stream of nitrogen.

Supplementary Section S8. Chemical derivatization

Dried extracts were derivatized by sequential methoximation and trimethylsilylation. Methoximation and trimethylsilylation were each performed for 60 min at 45°C using a Gerstel multipurpose autosampler.

Retention-index n-alkanes and the injection standard 4,4′-dibromooctafluorobiphenyl were added before instrumental analysis. Derivatization increased the volatility and chromatographic suitability of polar metabolites for gas chromatography–mass spectrometry.

Supplementary Section S9. GC–MS acquisition

Metabolomic profiling was performed using an Agilent 7250 gas chromatography/quadrupole time-of-flight mass spectrometer coupled to an Agilent 8890 gas chromatograph.

Chromatographic separation was performed using an Rxi-5ms capillary column. Electron-impact ionization was performed at 70 eV, and ions were acquired across a mass-to-charge range of m/z 50–550.

Analytical reference standards supported targeted compound identification and quantification. Untargeted annotation was based on spectral-library matching after peak detection, spectral deconvolution, retention-time alignment and feature alignment across samples.

Supplementary Section S10. Targeted data processing

Targeted metabolomic data were processed using Agilent MassHunter Quantitative Analysis version 10.2. Targeted compounds were identified and quantified using analytical reference standards, and peak integration was reviewed according to the targeted-processing workflow.

Annotations supported by analytical reference standards were treated as higher-confidence identifications than untargeted library-based annotations.

Supplementary Section S11. Untargeted data processing

Untargeted metabolomic data were processed using MS-DIAL version 4.9.2. The processing workflow comprised peak detection, spectral deconvolution, retention-time alignment, feature alignment across samples, spectral-library matching and tentative metabolite annotation. Targeted and untargeted peak lists were processed separately before integration.

Supplementary Section S12. Data integration and duplicate handling

The targeted and untargeted datasets were merged into a single analytical feature matrix. When both workflows annotated the same metabolite, the targeted signal supported by an analytical reference standard was retained, and the corresponding untargeted feature was removed before statistical modeling.

This procedure prevented duplicated metabolite identities while retaining metabolites detected exclusively by the untargeted workflow. The final integrated dataset contained 164 non-duplicated metabolite features.

Supplementary Section S13. Quality control and normalization

Metabolite features were normalized to the relevant isotopically labeled internal standards. The retained data matrix was subsequently quantile normalized across samples. Quantile normalization was used to harmonize the empirical abundance distributions across samples; it is not a direct batch-effect correction.

Features were retained for statistical analysis when both of the following quality-control criteria were satisfied:

- relative standard deviation <0.20; and
- D-ratio <0.40.

The relative standard deviation quantified technical variation in quality-control samples. The D-ratio represented technical variation relative to total observed variation and was used to exclude features for which analytical variation was large in relation to biological variation.

Supplementary Section S14. Data transformation and missing data

The integrated metabolite matrix was transformed as $\log_2(x+1)$. Adding 1 prevented undefined values when the measured abundance was zero. Of 13, 120 expected measurements, 34 (0.259%) were missing, all for cysteine (34/80; 42.5%): 6/20 at rest, 10/20 at the AT, 8/20 at peak and 10/20 during recovery. The missing cysteine measurements were not imputed and remained missing in the final modeling matrix**.** The other 163 metabolites were complete. Although the preprocessing code contained random forest imputation function, these cysteine values remained missing in the final modeling matrix; the cysteine-specific mixed-effects models therefore used available observations. Participant-level clinical variables and plasma glucose concentrations were not imputed.

All formal metabolomic modeling used the transformed feature matrix, with available observations for cysteine.

Supplementary Section S15. Descriptive metabolomic analyses

Descriptive metabolite abundances were calculated from the untransformed and non-imputed data to preserve the original analytical measurement scale. For each metabolite and workload, descriptive statistics are reported in Supplementary Table S1A.

Relative percentage changes from rest were calculated separately for the anaerobic threshold, peak exercise and recovery and are reported in Supplementary Table S1B. Percentage changes were used only for descriptive presentation; all inferential analyses were based on $\log_2(x+1)$-transformed values.

Supplementary Section S16. Clinical descriptive statistics and sample size

Categorical variables were summarized as n (%). Continuous variables were summarized as mean±SD, median (IQR), minimum and maximum.

The available sample size was determined by the number of eligible participants from the parent trials who completed the screening cardiopulmonary exercise test. No separate sample-size calculation was undertaken for this exploratory secondary analysis.

Supplementary Section S17. Linear mixed-effects modeling framework

Linear mixed-effects models were fitted separately for each of the 164 metabolites. Repeated measurements at rest, the anaerobic threshold, peak exercise and recovery were nested within participants.

Participant ID was included as a random intercept to account for within-participant correlation. Workload was modeled as a categorical fixed effect, with rest as the reference category. Consequently, workload coefficients represented the mean difference in $\log_2(x+1)$-transformed metabolite abundance at the anaerobic threshold, peak exercise or recovery relative to rest.

Models were fitted using maximum likelihood rather than restricted maximum likelihood to maintain a consistent estimation framework across models with different fixed-effect structures. Random-effect variance was treated as a nuisance parameter and was not interpreted as a study outcome. Satterthwaite's approximation was used to calculate denominator degrees of freedom and two-sided p values for fixed effects. Reported 95% confidence intervals were Wald confidence intervals.

Supplementary Section S18. Model specifications

The following models were fitted, where (1|ID) denotes the participant-specific random intercept:

S18.1. Model 0: unadjusted workload model

Value ~ Workload + (1|ID)

Model 0 estimated unadjusted workload differences at the anaerobic threshold, peak exercise and recovery relative to rest.

S18.2. Model 1: covariate-adjusted workload model

Value ~ Workload + Age + Sex + HbA1c + Diabetes duration + (1|ID)

Model 1 was the primary workload model and adjusted the workload coefficients for age, sex, HbA1c and diabetes duration.

S18.3. Model 2: workload × HbA1c interaction model

Value ~ Workload × HbA1c_c + Age + Sex + Diabetes duration + (1|ID)

HbA1c_c denotes mean-centered HbA1c. Model 2 examined whether workload-associated metabolite responses differed according to chronic glycemic control.

S18.4. Model 3: workload × resting plasma glucose interaction model

Value ~ Workload × Resting glucose_c + Age + Sex + HbA1c + Diabetes duration + (1|ID)

Resting glucose_c denotes mean-centered resting plasma glucose. Model 3 examined whether workload-associated metabolite responses differed according to acute glycemic status before exercise.

S18.5. Model 4: independent $\dot{V}O_2$peak association model

Value ~ Workload + $\dot{V}O_{2peak}$_c + Age + Sex + HbA1c + Diabetes duration + (1|ID)

$\dot{V}O_{2peak}$_c denotes mean-centered $\dot{V}O_{2peak}$. Model 4 assessed the independent association between cardiorespiratory fitness and metabolite abundance across the exercise protocol.

S18.6. Model 5: workload × $\dot{V}O_2$peak interaction model

Value ~ Workload × $\dot{V}O_{2peak}$_c + Age + Sex + HbA1c + Diabetes duration + (1|ID)

Model 5 examined whether the metabolite response to exercise differed according to cardiorespiratory fitness. Female sex was the reference category in all models containing sex.

Supplementary Section S19. Multiple-testing correction

P values were adjusted using the Benjamini–Hochberg false discovery rate procedure. Adjustment was performed separately within each model term across the 164 metabolites. Thus, the anaerobic-threshold, peak and recovery workload coefficients, covariate main effects and workload interaction coefficients were corrected within their respective statistical families.

A q value <0.05 was considered statistically significant. All BH-FDR-significant fixed effects identified across Models 0–5 are reported in Supplementary Table S2A.

Supplementary Section S20. Prediction analyses for significant $\dot{V}O_2$peak interactions

Prediction analyses were performed for metabolites with a BH-FDR-significant workload × $\dot{V}O_{2peak}$ interaction in Model 5. Fixed-effect workload-minus-rest contrasts were evaluated at the 10th and 90th percentiles of the observed $\dot{V}O_{2peak}$ distribution, corresponding to 17.4 and 35.8-ml kg^{-1} min^{-1}, respectively.

Age, HbA1c and diabetes duration were held at their sample means, and sex was held at the female reference category. Predictions were calculated on the $\log_2(x+1)$-transformed abundance scale.

For each fitness value, the contrast vector was defined as the Model 5 design row for the relevant workload minus the design row for rest. The contrast standard error was calculated from the fixed-effect variance–covariance matrix as $\sqrt{(c'Vc)}$, where c was the contrast vector and V was the covariance matrix. Wald 95% confidence intervals were calculated as the predicted contrast ±1.96 standard errors.

Predicted contrasts and confidence intervals are reported in Supplementary Table S2B and visualized in Figure 1.

Supplementary Section S21. Repeated-measures analysis of plasma glucose

Changes in plasma glucose across rest, the anaerobic threshold, peak exercise and recovery were evaluated using one-way repeated-measures ANOVA among participants with complete measurements at all four workloads.

The uncorrected univariate ANOVA result was reported, and no sphericity correction was applied. Pairwise workload differences were assessed using paired t tests, with the resulting p values adjusted using the Holm method.

Descriptive glucose concentrations are reported in Supplementary Table S3. The repeated-measures ANOVA and Holm-adjusted pairwise comparisons are reported in Supplementary Table S4.

Supplementary Section S22. Workload-specific metabolite changes for correlation analyses

For each participant and metabolite, workload-specific changes were calculated on the $\log_2(x+1)$-transformed scale as:

- ΔAT–Rest = anaerobic-threshold value minus rest value;
- ΔPeak–Rest = peak value minus rest value; and
- ΔRecovery–Rest = recovery value minus rest value.

For each contrast, correlations were restricted to metabolites with a BH-FDR-significant Model 1 coefficient for that same contrast. The analyses therefore included two metabolites for ΔAT–Rest, 16 metabolites for ΔPeak–Rest and 21 metabolites for ΔRecovery–Rest.

Supplementary Section S23. Pearson correlation analyses

Pearson correlation coefficients were used to examine associations between workload-specific metabolite changes and age, BMI, HbA1c, diabetes duration, $\dot{V}O_{2peak}$, peak power relative to body mass (W/kg), exercise duration to the anaerobic threshold and exercise duration to peak workload.

All tests were two-sided. A nominal p value <0.05 was used to identify exploratory associations. Because these analyses were exploratory, correlation p values were not adjusted for multiple testing.

Correlation heatmaps are shown for ΔPeak–Rest in Figure 2a, ΔRecovery–Rest in Figure 2b and ΔAT–Rest in Supplementary Figure S7. Complete coefficients and unadjusted p values are provided in Supplementary Table S5.

Supplementary Section S24. Metabolite annotation and broad metabolic-category assignment

KEGG compound identifiers and pathway information were retrieved using KEGGREST when a reliable metabolite-name match was available. For visualization and over-representation analysis, each of the 164 metabolites was assigned to one of nine mutually exclusive broad metabolic categories using KEGG annotation where available, supplemented by predefined biochemical classification.

Metabolites without a specific category assignment were retained and classified as other/unclassified. Assignment to a broad metabolic category did not imply that every metabolite had a valid KEGG compound identifier.

The resulting annotation table was used to visualize adjusted Model 1 coefficients by broad metabolic category and to perform workload-specific metabolic-category over-representation analyses. Adjusted coefficients are visualized for peak exercise in Figure 3, the anaerobic threshold in Supplementary Figure S9 and recovery in Supplementary Figure S8.

Supplementary Section S25. Metabolic-category over-representation analysis

Metabolic-category over-representation was assessed separately for the anaerobic threshold vs rest, peak exercise vs rest and recovery vs rest. Metabolites with a BH-FDR-significant Model 1 workload coefficient ($q<0.05$) in the relevant comparison constituted the significant-metabolite list. These lists contained two metabolites for the anaerobic threshold, 16 for peak exercise and 21 for recovery.

All 164 metabolites included in Model 1 constituted the background universe ($N=164$). For a given workload comparison and category:

- n was the total number of BH-FDR-significant metabolites in that comparison.
- K was the number of background metabolites assigned to the category.
- k was the observed number of significant metabolites assigned to the category; and
- nK/N was the expected number of significant metabolites under the null hypothesis.

Over-representation was tested using the upper tail of the hypergeometric distribution:

$$P(X \geq k) = \text{phyper}(k-1, K, N-K, n, \text{lower.tail=FALSE}).$$

Fold enrichment was calculated as $k/(nK/N)$. The analysis recorded K, k, the expected count, fold enrichment, the unadjusted p value and the BH-FDR-adjusted q value for each category.

P values were adjusted across the nine metabolic categories separately within each workload comparison. A q value <0.05 was considered statistically significant. Complete results are reported in Supplementary Table S6.

Supplementary Section S26. Software and package versions

Statistical analyses were performed using R version 4.3.2 (R Foundation for Statistical Computing, Vienna, Austria). Linear mixed-effects models were fitted using lme4 version 1.1-38 and lmerTest version 3.2-0. Model estimates and Wald confidence intervals were extracted using broom.mixed version 0.2.9.6. Data processing used dplyr version 1.1.4, tidyr version 1.3.2 and purrr version 1.2.1. Figures were generated using ggplot2 version 4.0.1, ComplexHeatmap version 2.18.0, circlize version 0.4.17, patchwork version 1.3.2 and ggrepel version 0.9.6. KEGG annotation was performed using KEGGREST version 1.42.0.

Supplementary Table Legends

Supplementary Table S1A. Descriptive statistics for raw targeted and untargeted metabolomics data prior to log transformation.

Supplementary Table S1B. Relative percentage changes in metabolite abundance between exercise stages.

Supplementary Table S2A. Significant fixed effects identified by linear mixed-effects models (M0–M5) following Benjamini–Hochberg false discovery rate (BH-FDR) correction.

Supplementary Table S2B. Model-predicted workload-minus-rest metabolite responses at low (10th percentile) and high (90th percentile) cardiorespiratory fitness. Predictions are presented as estimated contrasts with 95% confidence intervals. Fitness was represented by mean-centered $\dot{V}O_{2peak}$ values from Model 5. Positive values indicate higher metabolite abundance relative to rest. Values are reported on the $\log_2(x + 1)$-transformed abundance scale.

Supplementary Table S3. Descriptive statistics of plasma glucose concentrations across exercise workloads.

Supplementary Table S4. Holm-adjusted pairwise comparisons of plasma glucose concentrations across exercise workloads.

Supplementary Table S5. Pearson correlations between workload-specific metabolite changes and clinical characteristics, cardiorespiratory fitness, and glycemic control.

Supplementary Table S6. Over-representation analysis of broad metabolic categories among metabolites with BH-FDR-significant Model 1 workload effects.

Supplementary Figure Legends

Supplementary Figure S1. Overview of the graded incremental cardiopulmonary exercise test and blood-sampling protocol. Participants completed pre-test screening and baseline assessments after avoiding meals and accompanying insulin boluses for at least 2 h. Following a safety glucose check, the cardiopulmonary exercise test (CPET) comprised passive rest, a 20-W warm-up, graded incremental exercise until volitional exhaustion, a 20-W cool-down, and passive recovery. Venous blood samples were collected at baseline, at the start of the CPET, every 3 min during incremental exercise, at peak exercise, and at the end of recovery. The sample collected closest to the modeled anaerobic threshold (AT) was used for the AT analysis. Breath-by-breath gas-exchange measurements were recorded continuously throughout the CPET, and a final safety glucose check was performed before departure.

Supplementary Figure S2. Heatmap of the 25 unique metabolites with at least one BH-FDR-significant Model 1 workload coefficient ($q<0.05$; n=20). Colors represent row-standardized z scores calculated across the workload-specific mean $\log_2(x+1)$-transformed abundances at rest, the anaerobic threshold, peak exercise and recovery. Red indicates higher and blue indicates lower relative abundance within each metabolite; Rows were hierarchically clustered. AT, anaerobic threshold; BH-FDR, Benjamini–Hochberg false discovery rate.

Supplementary Figure S3. Forest plot of BH-FDR-significant adjusted Model 1 workload coefficients for the anaerobic threshold, peak exercise and recovery vs rest ($q<0.05$; n=20). Points represent adjusted coefficients on the $\log_2(x+1)$-transformed abundance scale, and horizontal lines represent 95% Wald confidence intervals. Circles indicate negative coefficients and triangles indicate positive coefficients. The plot contains two anaerobic-threshold, 16 peak and 21 recovery associations; a metabolite may appear in more than one section when significant in more than one contrast. AT, anaerobic threshold; BH-FDR, Benjamini–Hochberg false discovery rate.

Supplementary Figure S4. Volcano plot of adjusted Model 1 coefficients for the anaerobic threshold vs rest across all 164 metabolites (n=20). The x-axis shows the adjusted coefficient on the $\log_2(x+1)$-transformed abundance scale, and the y-axis shows $-\log_{10}(q)$. Squares indicate BH-FDR-significant positive coefficients ($q<0.05$), whereas circles indicate non-significant coefficients. The horizontal dashed line indicates $q=0.05$, and the vertical dotted line indicates a coefficient of zero. AT, anaerobic threshold; BH-FDR, Benjamini–Hochberg false discovery rate.

Supplementary Figure S5. Volcano plot of adjusted Model 1 coefficients for peak exercise vs rest across all 164 metabolites (n=20). The x-axis shows the adjusted coefficient on the $\log_2(x+1)$-transformed abundance scale, and the y-axis shows $-\log_{10}(q)$. Squares and triangles indicate BH-FDR-significant positive and negative coefficients, respectively ($q<0.05$); circles indicate non-significant coefficients. The horizontal dashed line indicates $q=0.05$, and the vertical dotted line indicates a coefficient of zero. BH-FDR, Benjamini–Hochberg false discovery rate.

Supplementary Figure S6. Volcano plot of adjusted Model 1 coefficients for recovery vs rest across all 164 metabolites (n=20). The x-axis shows the adjusted coefficient on the $\log_2(x+1)$-transformed abundance scale, and the y-axis shows $-\log_{10}(q)$. Squares and triangles indicate BH-FDR-significant positive and negative coefficients, respectively ($q<0.05$); circles indicate non-significant coefficients. The horizontal dashed line indicates $q=0.05$, and the vertical dotted line indicates a coefficient of zero. BH-FDR, Benjamini–Hochberg false discovery rate.

Supplementary Figure S7. Exploratory Pearson correlations between metabolite changes from rest to the anaerobic threshold and clinical and exercise characteristics (n=20). The analysis was restricted to the two metabolites with a BH-FDR-significant Model 1 coefficient for the anaerobic threshold vs rest. Metabolite changes were calculated on the $\log_2(x+1)$-transformed abundance scale. Colors represent Pearson correlation coefficients. *$p<0.05$, **$p<0.01$ and ***$p<0.001$, based on unadjusted two-sided p values. AT, anaerobic threshold; BH-FDR, Benjamini–Hochberg false discovery rate; VO_{2peak}, peak oxygen uptake.

Supplementary Figure S8. Broad metabolic-category classification of metabolites with BH-FDR-significant Model 1 coefficients for recovery vs rest ($q<0.05$; n=20). Bars represent adjusted coefficients on the $\log_2(x+1)$-transformed abundance scale; positive and negative coefficients indicate higher and lower abundance during recovery, respectively. Colors indicate broad metabolic categories assigned using KEGG annotation where available, supplemented by predefined biochemical classification; metabolites without a specific category assignment were classified as other/unclassified. BH-FDR, Benjamini–Hochberg false discovery rate; KEGG, Kyoto Encyclopedia of Genes and Genomes; TCA, tricarboxylic acid.

Supplementary Figure S9. Broad metabolic-category classification of metabolites with BH-FDR-significant Model 1 coefficients for the anaerobic threshold vs rest ($q<0.05$; n=20). Bars represent adjusted coefficients on the $\log_2(x+1)$-transformed abundance scale; positive coefficients indicate

higher abundance at the anaerobic threshold. Colors indicate broad metabolic categories assigned using KEGG annotation where available, supplemented by predefined biochemical classification; metabolites without a specific category assignment were classified as other/unclassified. AT, anaerobic threshold; BH-FDR, Benjamini–Hochberg false discovery rate; KEGG, Kyoto Encyclopedia of Genes and Genomes.
